# Second Generation of HIV Surveillance System: A Pattern for Iran

**Published:** 2012-05-30

**Authors:** M Nematollahi, N Khalesi, H Moghaddasi, M Askarian

**Affiliations:** 1 Department of Health Information Management, Shiraz University of Medical Sciences, Shiraz, Iran; 2 Department of Health Services management, Iran University of Medical Sciences, Tehran, Iran; 3 Department of Health Information Management, Shahid Beheshti University of Medical Sciences, Tehran, Iran; 4 Department of Community Medicine, Shiraz University of Medical Sciences, Shiraz, Iran

**Keywords:** HIV/AIDS, Surveillance system, Iran

## Abstract

**Background:**

For the purpose of minimizing the HIV/AIDS epidemic effects, one of the programs is the promotion of scientific methods and setting of the suitable surveillance systems. The present research was conducted to design the HIV/AIDS surveillance system in Iran applying WHO recommendations and the experience of some countries.

**Methods:**

In 2009, based on the country's requirements, the HIV/AIDS surveillance system was proposed and designed for Iran. The Delphi technique was utilized to find the views of experts. Data analysis was conducted based on a comparison of the attributes of the HIV/AIDS surveillance systems in the countries under consideration using a descriptive and theoretical analysis.

**Results:**

The model was approved obtaining the final score of 36.3 out of 44, viz 82.5%.

**Conclusion:**

Designing and performing of the HIV/AIDS surveillance pattern in the direction of “second generation of HIV/AIDS surveillance” can be considered as an important step in the improvement of the patient’s control and precaution of HIV/AIDS.

## Introduction

For the purpose of minimizing the HIV/AIDS epidemic effects, one of the programs is the promotion of scientific methods and setting of the suitable surveillance systems.[1] The need was accomplished by World Health Organization (WHO) and The Joint United Nations Program on HIV and AIDS (UNAIDS), and was introduced as the “Second Generation HIV Surveillance System”.[2]

HIV Surveillance has been defined as the data collection discipline, interpretation and analysis of data; and releasing of information about the infected ones[3][4] and the terms such as HIV surveillance and HIV surveillance system are considered as the whole process of data collection and HIV informing as a whole.[5] It should be done continuously6 and the obtained results should be used for designing, performing, assessment of interventions and the health programs.[5]

The present research has been conducted to design the HIV/AIDS surveillance system in Iran applying WHO recommendations and the experience of the countries having the lead in the design and implementation of the system.

## Materials and Methods

In a descriptive applied study, in analysis stage of the current situation of HIV/AIDS surveillance in Iran, visiting the “center of diseases management”, interviewing and studying of forms and application of check lists in comparison with the data collection procedure was taken action. In the designing and performing of the system, some of the hygienic leading countries were chosen. Data collection pattern in the method of HIV/AIDS surveillance in these countries was based on studying and reviewing of the texts and the related sources.

In credit evaluation stage, the investigator’s suggested pattern, and opinions of eight experts who had the PhD degree in “Health Information Management”, and 14 specialists of the infectious diseases center’s opinions, who were working in HIV/AIDS research center in Imam Khomeini Hospital, were considered through Delphi technique. For experts and specialists selection, the random sampling method was used.

Validity of the related tools was modified through the content validity method (Correlation coefficient=0.87). According to the study of HIV/AIDS surveillance’s common and difference points in the selected countries (USA, UK, Australia, and Malaysia), and with the consideration of country’s need and requirements, HIV/AIDS surveillance has been presented in a frame with 4 patterns including data sources, data elements, data collection and reporting disciplines and data processing disciplines. Delphi technique was performed just because of its adjustment proportional to the situation of the country. In this stage, a questionnaire that included all of the above mentioned 4 patterns, and had 35 questions and compared the opinions of experts, was distributed among them.

## Results

Although an HIV/AIDS surveillance system was present in Iran, but HIV/AIDS surveillance management discipline was not in the direction of”second generation HIV surveillance system” approved by WHO. Data sources in the HIV/AIDS surveillance system were based on the suggested patterns of biologic surveillance, behavioral surveillance, HIV/AIDS reporting system (as main sources) and sexual transmitted infections surveillance, tuberculosis surveillance, hepatitis surveillance and death registry (as ancillary sources)

The results of Delphi technique and final patterns in Iran were presented in Tables 1, 2, 3.

**Table 1:** Data elements of main sources.

**Data source **	**Type**
**Biologic surveillance**	Age; gender; educational, economical and social situation; residential or immigration situation; delivering (for the pregnancy centers); marital status
**Behavioral surveillance**	Hazardous behaviors that can cause epidemic; sub-population who have the hazardous behaviors, size of sub-populations; amount of HIV infection in the above mentioned sub-populations; type and amount of relationship between hazardous sub-population and the whole ones
**HIV/AIDS reporting system**	Demographic data, medical data, complementary data

**Table 2:** Data collection and reporting disciplines.

**Subcategory**	**Comment**
**Methods of data collection**	• Active data collection
	• passive data collection
**Reporting disciplines**	• For the reporting of HIV/AIDS cases, special forms should be used by the physicians.
	• The approving of punishment regulation, for those who are avoiding the report of HIV/AIDS cases is necessary.
**Linkage with other systems**	• The collation of HIV/AIDS data with the other systems -such as sexual transmitted diseases, tuberculosis and hepatitis surveillance systems and national index of mortality rate- is necessary.
**Confidentially**	• Approval of HIV/AIDS data’s regulations confidentially.
	• Saving of unnecessary electronic reports and paper ones to the bare minimum amount.
	• Name omission from those documents that do not preserve for the hygienic purposes.
	• Preserving those documents which have the data, in a secure place and application of password and computer encoding.
	• Application of HIV/AIDS data for the investigation purposes should be approved by the responsible people and researchers should sign the declaration confidentially.
	• Application of name and surname transferring system to the soundex, in the case of data transferring process.
	• Data transferring through document gateway or electronics post with the use of encoding excel.

**Table 3:** Data process disciplines in the HIV /AIDS surveillance system based on the suggested

**Pattern category**	**Comment**
**Race groups**	Kurd, Lore, Balouch, Turk, Persian, Gilaki, Arab, Turkaman , others ****
**Age groups**	• <13
	• 13-14
	• 15-19
	• group which includes 5 to 64 years old
	• 65 +
	• unspecified
**Infection rate’s classification **	≥13 years old: Women (injection addiction, hazardous heterosexual sex, blood or tissue reception, and the others).
	Men (as Women+MSM, MSM and injection addiction).
	• <13: Hemophilia/coagulation disorders, was born from the infected mother, blood or tissue reception, others.

## Discussion

For diseases such as AIDS, the convincing statistics are needed for the purpose of HIV prevalence’s estimation and also the estimation of its social and economical expenses. These challenges the need for improvement of the surveillance system’s and theoretical equipments.[7] HIV/AIDS surveillance system in Iran is not in the direction of “second generation of HIV/AIDS surveillance system”. This system that has been recommended by WHO, helps to collect data sources (that has provided useful information for the purpose of HIV outbreak’s deduction, and controlling of the infected ones).[2][8] Developing countries face several challenges to implement surveillance programs.[9] Most of developing countries have collected few information about phases of the disease, its rate of occurrence, the rate of clinical tolerance, and the number of mortality rate, and the behavioral researches.[10]

Data sources HIV/AIDS surveillance system in Iran is not comprehensive. In USA, CDC recommends that all states and territories to conduct case surveillance for human immunodeficiency virus infection as an extension of current acquired immunodeficiency syndrome surveillance activities.[11] In UK, the HIV and STIs Department utilizes a number of data sources for their surveillance systems.[12]

According to the suggested pattern, data element should include biological, behavioral, demographic, medical and supplementary data. Australian HIV Public Access Data Set include state, sex, year of HIV diagnosis, HIV exposure category, year of last negative HIV antibody test, year of HIV seroconversion illness and CD4 count at HIV diagnosis.[13] In UK, behavioral data include year of first sex, pregnancy history, likely route of infection, and location of infection (heterosexual), sexual orientation, number of partners, sex abroad, concurrent STI and previous gonorrhea.[14]

In HIV/AIDS data processing, age and race grouping and risk classification should be done accurately. In Iran, there are no race and age grouping and risk classification are not appropriate. In the selected countries, age and race grouping and risk classification are completely done.[13][14][15][16] According to the suggested pattern, HIV/AIDS surveillance system should be linked with sexual transmitted diseases, tuberculosis and hepatitis surveillance systems. There is a correlation in the selected countries.[13][14][15][16] The second generation surveillance system has also stressed the necessity of this relationship.[2]

According to the suggested pattern, data confidentiality is very important in the HIV/AIDS surveillance system. In Iran, reported cases to Diseases Management Center are associated with the name and address, so privacy is marred. In the selected countries, they use soundex code for data reporting. In USA, most states have specific statutory protections for public health data related to HIV infection and other STDs. However, state legal protections vary and CDC supports additional efforts to strengthen privacy protections for public health data. CDC has recommended additional standards to enhance the security and confidentiality of HIV and AIDS surveillance data.[11]

Considering the HIV outbreak in Iran, designing and performing of the HIV/AIDS surveillance pattern in the direction of “second generation of HIV/AIDS surveillance” can be considered as an important step in the improvement of the patient’s controlling and precaution of HIV/AIDS.

## References

[1] (2005). Guidelines for measuring national HIV prevalence in population-based surveys. UNAIDS/WHO.

[2] (2000). Guidelines for second generation HIV surveillance. World Health Organization and the Joint United Nations Programme on HIV/AIDS, WHO/ UNAIDS.

[3] (2008). Monitoring the Declaration of Commitment on HIV/AIDS: Guidlines on construction of core indicators, UNAIDS.

[4] (1998). Surveillance of communicable diseases: a training manual.

[5] Family Health International, Behavioral Surveillance Survey in the Highway Route of Nepal: Round No.3, Report 2001.

[6] Jekel J (2007). Epidemiology, Biostatistics and Preventive Medicine. 3rd ed.

[7] Garrett GP, Grassly NC, Boerma JT, Ghys PD (2004). Maximizing the global use of HIV surveillance data through the development and sharing of analytical tools.. Sex Transm Infect.

[8] McDonald AM, Gertig DM, Crofts N, Kaldor JM (1994). A national surveillance system for newly acquired HIV infection in Australia. National HIV Surveillance Committee.. Am J Public Health.

[9] Rennie S, Turner AN, Mupenda B, Behets F (2009). Conducting Unlinked Anonymous HIV Surveillance in Developing Countries: Ethical, Epidemiological, and Public Health Concerns.. PLoS Med.

[10] Diaz T, De Cock K, Brown T, Ghys PD, Boerma JT (2005). New starategies for HIV surveillance in resource-constrained setting: an overview.. AIDS.

[11] (1999). Guidelines for National Human Immunodeficiency Virus Case Surveillance, Including Monitoring for Human Immunodeficiency Virus Infection and Acquired Immunodeficiency syndrome. Centers for Disease Control and Prevention (CDC).. MMWR Recomm Rep.

[12] Dougan S, Payne LJ, Brown AE, Fenton KA, Logan L, Evans BG, Gill ON (2004). Black Caribbean adults with HIV in England, Wales, and Northern Ireland: an emerging epidemic?. Sex Transm Infect.

[13] National Centre in HIV Epidemiology and Clinical Research (NCHECR). Surveillance.. http://www.nchecr.unsw.edu.au/NCHECRweb.nsf/page/home.

[14] HIV and STIs Surveillance Systems, Health Protection Agency. http://www.hpa.org.uk/HPA/Topics/InfectiousDiseases/InfectionsAZ/1202115502759.

[15] HIV/AIDS Statistics and Surveillance. Centers for Disease Control and Prevention (CDC).. http://www.cdc.gov/hiv/topics/surveillance/index.htm.

[16] Malaysian AIDS Council. 2009 Annual Report.. www.mac.org.my/v2/wp-content/uploads/attachment/annual09.pdf.

